# Natural and Induced T Regulatory Cells in Cancer

**DOI:** 10.3389/fimmu.2013.00190

**Published:** 2013-07-11

**Authors:** Dennis O. Adeegbe, Hiroyoshi Nishikawa

**Affiliations:** ^1^Experimental Immunology, Immunology Frontier Research Center, Osaka University, Suita, Japan

**Keywords:** Tregs, Foxp3, natural, induced, cancer, tumor, Interleukin-10, transforming growth factor β

## Abstract

CD4+Foxp3+ T regulatory (Treg) cells control many facets of immune responses ranging from autoimmune diseases, to inflammatory conditions, and cancer in an attempt to maintain immune homeostasis. Natural Treg (nTreg) cells develop in the thymus and constitute a critical arm of active mechanisms of peripheral tolerance particularly to self antigens. A growing body of knowledge now supports the existence of induced Treg (iTreg) cells which may derive from a population of conventional CD4+ T cells. The fork-head transcription factor (Foxp3) typically is expressed by natural CD4+ Treg cells, and thus serves as a marker to definitively identify these cells. On the contrary, there is less consensus on what constitutes iTreg cells as their precise definition has been somewhat elusive. This is in part due to their distinct phenotypes which are shaped by exposure to certain inflammatory or “assault” signals stemming from the underlying immune disorder. The “policing” activity of Treg cells tends to be uni-directional in several pathological conditions. On one end of the spectrum, Treg cell suppressive activity is beneficial by curtailing T cell response against self-antigens and allergens thus preventing autoimmune diseases and allergies. On the other end however, their inhibitory roles in limiting immune response against pseudo-self antigens as in tumors often culminates into negative outcomes. In this review, we focus on this latter aspect of Treg cell immunobiology by highlighting the involvement of nTreg cells in various animal models and human tumors. We further discuss iTreg cells, relationship with their natural counterpart, and potential co-operation between the two in modulating immune response against tumors. Lastly, we discuss studies focusing on these cells as targets for improving anti-tumor immunity.

## Introduction

Early studies of T regulatory (Treg) cells, defined as a subset of CD4+ cells that co-express high levels of CD25, the high affinity IL-2 receptor α-chain, demonstrated unequivocally that these cells are crucial for maintenance of peripheral self tolerance as their elimination led to development of multiple organ-specific autoimmune diseases (1). Subsequent studies identified foxp3, a member of the fork-head/winged-helix family of transcriptional factor as uniquely expressed by Treg cells and allowed for more precise phenotypic identification of these cells as CD25 alone was insufficient due to its upregulation on activated T cells (1–3). Endowed with highly suppressive machinery, it is now well established that CD4+Foxp3+ Treg cells regulate a diverse array of immune responses ranging from autoimmune disease, allergies, and transplant rejection, to infections and cancers (4). While generally beneficial in the former conditions, the inhibitory activity of Treg cells often antagonizes protective immunity in the latter settings. Depending on the microenvironment in which they are found, and potential stimuli eliciting their recruitment or presence at such sites, CD4+Foxp3+ Treg cells are now broadly described as natural or adaptive (5, 6). Natural CD4+Foxp3+ Treg cells are the better understood of the two with the central dogma being that the adaptive or “induced” cells are generally derived from existing pool of naïve conventional CD4+ T cells. Regardless of their origin, they share one key feature: their ability to potently suppress effector T cells (5). Although expression of Foxp3 generally identifies natural, thymus-derived CD4+ Treg cells, adaptive Treg cells may or may not express this transcription factor (5, 7, 8).

Recent years have seen a surge in studies of cancer models and in humans highlighting the elevated levels of Treg cells in the tumor and/or in circulation (9, 10). This often correlates with poor anti-tumor effector response, hence compromised tumor immunity (11, 12). Whether the Foxp3+ cells widely described in many cancer settings are of natural or adaptive/induced type remains largely a bone of contention. This review focuses on the current knowledge about both subsets of Treg cells, their generation, phenotypic characteristics, and ill-defined roles as described in various tumor models and human cancers. Current therapeutic modalities geared toward Treg depletion and how they may impinge on recruited natural versus tumor-induced Treg (iTreg) cells are discussed.

## Induced/Adaptive Tregs, More than Just Foxp3+ Cells

Adaptive Tregs encompass a number of CD4+ cells with regulatory/suppressive capabilities (7, 8, 13). Although “iTregs” is commonly used interchangeably with “adaptive Tregs,” the former is perhaps a better nomenclature for all extrathymically derived CD4+ Treg cells. In this context, iTreg cells range from Tr1 cells, which are induced by IL-10, and secrete both IL-10 and TGF-β (7), to TGF-β-producing Th3 cells (induced by oral antigen tolerizing conditions) (8), to peripheral naïve CD4+CD25−Foxp3−cells that become converted to Foxp3-expressing cells (13). Tr1 cells regulate immune responses against ubiquitous commensal organisms and promote tolerance in the gut, and accumulating evidence reveal they play key roles in other facets of adaptive immune response (7). Th3 cells on the other hand, appear critical in tolerance induced by oral antigen delivery (8). Both adaptive Treg cell types are induced in peripheral sites and have been described to generally lack expression of Foxp3 which distinctively identifies natural Treg (nTreg) cells of thymic origin (2, 3, 14). In most tumor studies however, these cells have not been extensively described. Most of the attention on iTregs in tumor settings has largely focused on converted Foxp3-expressing cells mentioned above. Since both peripherally induced Foxp3+ as well as Foxp3 non-expressing CD4+ regulatory T cells (e.g., Tr1− cells) are often discussed under the umbrella of “induced” Treg cells, for simplicity sake, the term iTreg in this review will be restricted to CD4+CD25−Foxp3−cells that have acquired Foxp3 expression. In order to do justice to their contributions in tumor settings, other Foxp3 non-expressing, peripherally induced CD4+ regulatory cells, specifically Tr1 cells, will be discussed separately as such in one section and the rest of our discussion will focus on Foxp3+ peripherally converted iTregs.

## Natural Versus Induced Tregs in Cancer: Origin and Accumulation

Several lines of evidence reveal an accumulation of Treg cells both at peripheral sites (spleen, peripheral blood), and within the local tumor microenvironment [reviewed in Ref. (10, 12, 15)]. This often correlates with persistent tumor burden and poor anti-tumor effector response (11, 12). Importantly, a low CD8+ effector T cell number is also noted relative to the high proportion of Foxp3+ Treg cells in the peripheral blood and tumor tissue in many cancer patients (12) suggesting active recruitment of Foxp3+ Treg cells is a key feature of many tumors. Thus, a “guilty-by-association” analogy means that these tumor-infiltrating Treg cells must at least, in part, be responsible for dampening anti-tumor immunity, namely preventing effective tumor immunosurveillance. One outstanding issue however is the source of these cells, and this issue is currently a subject of debate within the tumor immunology community.

From current knowledge, the composition of Foxp3+ Treg cells within tumors and/or in circulation in human cancer patients remains poorly understood. There are a few possibilities: (1) They are nTregs recruited to the tumor site and actively expanding (16–18); (2) They are a pool of induced, Foxp3-acquired Treg cells (iTregs) derived from converted CD25− cells (19, 20); (3) They are Tr1 cells (discussed in the following section). In support of the first possibility, studies performed by Zou and colleagues demonstrated specific recruitment of pre-existing human Treg cells into tumors in a manner that was dependent on tumor-mediated CCL22 production and gradient (16). Another study demonstrated that Treg cells underwent substantial proliferation at tumor site and draining lymph node in response to TGF-β secreted by immature DCs which themselves were a result of tumor cell modification (18). In either study however, the possibility that iTregs were also recruited or expanded at tumor site could not be excluded. The notion that tumor-infiltrating Tregs are likely expanded nTreg cells was further purported in a study that examined the TCR repertoire analysis of tumor-infiltrating Treg and T conventional cells (17). In this report, authors concluded that since the TCR repertoires of either population were largely non-overlapping, the tumor-infiltrating Tregs are likely of natural origin as a significant overlap would have been observed if a fair amount of CD25− cell conversion to Foxp3+ cells occurred.

Data supporting the second possibility comes from a number of studies (19–21). One of these demonstrated that in thymectomized, and anti-CD25-treated tumor-bearing mice, a population of Treg cells converted from CD25− cells developed (20). Anti-CD25 Treg depletion strategy has been described not to efficiently eliminate Treg cells (22). So the possibility remains that nTreg cells not touched by the treatment regimen expanded in this system. In any case, the thymectomy would have at least reduced any potential contribution by newly generated nTreg cells after anti-CD25 treatment cessation. Many tumors secrete TGF-β that may directly or indirectly induce naïve T cell conversion to Foxp3+ iTregs (19, 20, 23) Consistent with this, another group demonstrated that in a mouse prostate tumor model, tumor-derived TGF-β potentiated the conversion of CD4+CD25− T conventional cells into Foxp3-expressing, CD25+ iTreg cells (19). However, sole presence of iTreg or nTreg cells within the tumor need not be mutually exclusive as demonstrated by Zhou et al. Using an influenza hemagglutinin (HA)-expressing tumors along with HA TCR-transgenic T cells in an adoptive transfer system, they were able to demonstrate that both *de novo* generated adaptive and nTreg cells contributed to the pool of tumor-Treg cells (24). Thus, a more realistic view of their composition is that both adaptive and nTreg cells contribute to the total Treg pool affiliated with tumor microenvironment.

## Tr1 Cells in Cancer

Not all regulatory CD4+ cells are endowed with Foxp3 suppressive machinery. As mentioned previously, IL-10-producing Tr1 cells fall under this umbrella of Foxp3-non-expressing cells. Tr1 cells by their original description in the early literature are CD4+CD25−, IL-10, and TGF-β-producing cells (7). The general consensus is that they are derived from a pool of naïve CD4+ T cells that are distinct from thymus-derived Foxp3+ cells. Suffice to say, they are seemingly low in frequency in an unperturbed immune environment but are readily detected in an environment rich in cytokines such as IL-10, justifying their label as adaptive or induced regulatory T cells.

Unlike CD4+Foxp3+ Treg cells, the involvement of Tr1 cells in tumors has not received as much attention. There are a number of studies showcasing the importance of these cells in tempering anti-tumor response, some dating back to pre-Foxp3 years (25–30). In a cohort of Hodgkins lymphoma patients, an argument was made by Marshall and colleagues for a contributory role of CD4+ IL-10+ Tr1 cells toward ineffective clearance of Hodgkins lymphoma. This was in part based on their finding that these cells were present at elevated proportions in associated lymph nodes, and could suppress T cell response in corresponding PBMCs (26). The co-existence of the Tr1 cells with CD4+CD25+ (presumably natural Foxp3+) both of which were enriched in the lymph nodes in this particular study makes it difficult to ascertain to what extent, if any, the Tr1 cells played an inhibitory role. Whiteside and colleagues have reported extensively the presence of Tr1 cells in head and neck squamous-cell carcinoma (HNSCC) patients (10). Although relatively low in frequency in circulation, they were present in a sizable proportion in tumor-infiltrating lymphocytes (28). *In vitro* analysis of peripheral CD4+ cells in glioblastoma patient also revealed a prominent Tr1 response against tumor cells suggestive of an enriched population of Tr1 cells in this setting (27). In a protocol involving adoptive transfer of *in vitro*-cultured Th1-like cells to ovarian cancer patients, Tr1 cells were also shown to contribute to the total circulating Treg pool (30). In general, many of the analyses performed in these studies were dependent on stimulation of patient’s PBMC with or without tumor antigens plus Tr1 cell-enhancing cytokines to showcase their existence, and demonstrate that cancer patients harbor more Tr1 cells than healthy individuals. Perhaps, most of the Tr1 cells in the periphery exist in precursor form and are only expanded at tumor site where antigen is ubiquitous and key cytokines such as IL-10 are abundant, similar to the *in vitro* simulations. The study performed by Bergmann et al., certainly is in agreement with this notion (28).

The mechanisms by which Tr1 cells might be induced within the tumor remains unclear. Some lines of evidence suggest that certain factors uniquely produced by tumor cells could facilitate an IL-10-rich environment that ultimately fosters Tr1 cell induction (10, 27). In one report, cyclooxygenase-2 (COX-2) overexpressing glioma via Prostaglandin E2 (PGE2) synthesis induced mature DCs to express high levels of IL-10, which in turn induced CD4+ T cells that secreted copious amounts of IL-10 and TGF-β (27). Furthermore, CD4+ T cells isolated from peripheral blood of glioblastoma patient showed marked IL-10 production against tumor cells indicating an enrichment of Tr1 cells within the peripheral CD4+ T cell pool in this patient. This sentiment was echoed by another study which demonstrated that *in vitro*, highly suppressive Tr1 cells were generated from CD4+CD25− T cells in the presence of autologous DCs and irradiated COX-2+ HNSCC cells or exogenous PGE2, with a cytokine cocktail that included IL-10 (29). Like the afore-mentioned study, the overall conclusion here is that COX-2 overexpression, and PGE2 production by HNSCC plays a key role in the induction of Tr1 cells in this malignancy. The Tr1 cells in this study however, were shown to have some Foxp3 expression.

One important point is that a unifying phenotype that definitively identifies these CD4+ Tr1 cells is yet to be agreed upon. Besides being CD25 negative, IL-10, and TGF-β-producing, their Foxp3 status remains a divisive subject. Some studies showed they express variable Foxp3 levels (28, 29, 31), others described them as Foxp3 negative, or foxp3 status was not addressed (26, 27, 30, 32, 33). The differences between these studies may likely stem from experimental designs although it can be argued that the stimulatory conditions used in some of the *in vitro* assays to amplify Tr1 cells are also conducive to Foxp3 induction in lieu of the fact that conventional human T cells can upregulate FOXP3 upon activation (34). Regardless of how they are described, Tr1 cells, like their natural counterparts, are capable of exhibiting potent suppressive functions as demonstrated in some of the above-mentioned studies.

With respect to their perceived function within the tumor microenvironment, it remains a possibility that they co-operate with nTregs, a notion that has been suggested by others (35). The dichotomy that Tr1 cells are increased in frequency in advanced cancer stage and also in patients who had no evidence of active disease following oncologic treatments when compared with early stage raises the possibility that they may play differing roles under varying tumor burdens (28). On the far end of the spectrum of possibilities is that Tr1 cells actually may play beneficial roles that are masked by the over-representation of their “natural cousins” within the tumor microenvironment. Perhaps the ratio between nTregs and Tr1 iTregs may be key to understanding their contribution to shaping the course of tumor progression. In support of this idea, *ex vivo* stimulated PBMCs of ovarian cancer patients who had better survival outcomes upon previous infusion with Th1-like CD4+ cells, contained higher fractions of both CD4+CD25+CD45RO+FoxP3+ and CD4+CD25−FoxP3−IL-10-producing cells compared to cells derived from short-term survivors (30). Importantly, the ratio of the Foxp3+ nTregs versus IL-10+ Tr1 cells was touted to be key to better outcome as the one patient that remained cancer-free showed a dwindling pattern in the frequency of CD4+Foxp3+ cells while the Tr1 cell numbers steadily increased with each cycle of T-cell infusion and *ex vivo* PBMC stimulation. Could induced regulatory cells that present in the form of IL-10-producing Tr1 cells be beneficial in the context of tumor immunity? Perhaps some studies in the foreseeable future may specifically tackle this question. IL-10 being a cytokine that appears to play both inhibitory and immunostimulatory roles (25, 26, 32, 36), an anti-tumor immunity-boosting role for IL-10+ Tr1 cells is thus, not unimaginable and the above study certainly leaves room for such deduction. Consistent with this notion, IL-10-producing CD4+ cells have been demonstrated to effect tumor rejection in a murine glioma model by augmenting CTL and NK cell response (32). Perhaps, “curative” outcome seen from a combination of standard cancer treatments and immune modulatory protocols favor an increase in a discrete, unobstructive, Tr1 cell population with a concomitant decrease in a tampering nTreg subset. At any rate, more studies are warranted to better understand how Tr1 cells shape the course of anti tumor immunity, and by extension, tumor progression. In addition, identification of reliable markers to pin-point categorically their existence in tumor mass and in circulation of cancer patients without a need to amplify them *in vitro* is necessary.

## Differentiating Natural Tregs from Induced Tregs

### Helios

Expression of Helios, a member of the Ikaros transcription factor family has been described to be a part of Treg genetic signature based on a number of gene array analysis (37, 38). In a recent report, essentially all thymic Treg cells were Helios+ but only about 70% of the peripheral pool retained their expression (39). Furthermore, *in vitro* and *in vivo*-generated iTregs failed to express Helios. An argument was thus made that Helios expression may mark the bona fide nTregs of thymic origin (39). Building on this observation, studies in tumor-bearing mice and human cancers have also explored the composition of tumor-infiltrating Treg cells with respect to Helios expression (40–42). Treg cells from peripheral blood of renal cell carcinoma (RCC) patients were found to consist of a population that expressed Helios (40). In human ovarian carcinomas, CXCR3+ Treg cells were reported to be abundantly represented in the majority of tumor-Treg cells and they co-express Helios (41). In another study that used a xenogeneic mouse model of malignant human brain tumor, it was demonstrated that majority of tumor-associated Treg cells expressed Helios, and their frequency decreased when tumor-bearing mice were thymectomized prior to tumor cell implantation (42). In all of these studies, the consensus was that the Treg cells within the tumors are most likely natural due to their expression of this transcription factor. On the contrary, it was reported that the vast majority of tumor-infiltrating Treg cells in a murine colon adenocarcinoma expressed low levels of Helios and the authors concluded that based on this phenotype, coupled with additional markers, these are likely to be iTregs (43). In the absence of any immune pathology in the colon however, it should be pointed out that colonic Treg cells may be predominantly thymus-derived nTreg cells as recently demonstrated (44). When weighed together, these observations only reinforce the possibility that the expression of Helios on tumor-infiltrating Treg cells may not necessarily be an indication that they are derivatives of nTreg cells. Further putting into question the reliability of Helios in resolving the dichotomy of “i” versus “n” Treg cells are some existing reports (45–47). Using polyclonal or antigen-specific stimulation methods to activate T cells derived from TCR-transgenic Rag^−/−^ mice (hence, no endogenous Tregs), Wraith and colleagues demonstrated that a substantial fraction of *in vitro*-generated iTregs expressed Helios under the latter stimulation condition (47). Another group also described transient expression of Helios on activated human and murine T conventional and Treg cells (45). Whether Helios positive versus negative Foxp3+ cells simply represent different versions of the same Treg group (i.e., n Tregs) is of particular interest given that the profile of iTreg cells generated in adoptively transferred lymphopenic mice based on gene expression analysis was found to be relatively similar to nTreg cells from normal mice (48). As Treg cells encounter tumor-associated antigens (TAA), it remains a possibility that they become activated and upregulate Helios expression. In this context, expression of Helios simply is not sufficient to distinguish the origin of tumor-Tregs.

### Neuropilin-1

Neuropilin-1 (Nrp-1), a type-1 transmembrane protein is yet another molecule that is being implicated in the iTreg versus nTreg identification issue (43, 49, 50). Using microarray analysis, Haribhai and team demonstrated that iTreg cells induced *in vitro* under TGF-β and IL-2 expressed very low levels of *Nrp1* compared to nTregs cells (49). In an MBP-specific TCR-transgenic mouse model under Rag deficiency background, another report demonstrated the existence of Foxp3+ iTreg cells in peripheral compartments, which persisted even in athymic mice suggesting that they were extrathymically derived (50). These cells expressed low levels of Nrp-1. In a model of iTreg cell generation via mucosal routes, Lafaille and colleagues demonstrated that mucosal iTreg cells or iTreg cells generated *in vivo* under non-inflammatory conditions also express low levels of Nrp-1 unlike nTreg cells in which high expression levels were noted. Under inflammatory conditions however, iTreg cells upregulated its expression (43). In tumor settings, there is only scant data describing Nrp-1 expression in association with sub-phenotypes of Treg cells. In one report, there was a positive trend toward increased presence of a sizable fraction of Foxp3+ cells which exhibited low expression levels of Nrp-1 in the tumor tissue of tumor-bearing mice. In contrast, Nrp-1hi cells predominated in the spleen suggesting that the Nrp-lo phenotype may represent a population of iTreg cells induced locally within the tumor (43). Taken together, these studies allude to the possibility that Nrp-1 expression may be a good indicator for distinguishing between peripherally induced adaptive Treg cells and may be particularly suitable in deciphering the composition of tumor-infiltrating Foxp3+ Treg cells.

### Other markers

Worth mentioning are a myriad of cell surface molecules and receptors that have also been associated with tumor-infiltrating Treg cells (41, 51–56). Garpin (GARP; glycoprotein A repetitions predominant) was found in one study to be significantly higher on Foxp3+ Treg cells in hepatocellular carcinoma patients (55). Lymphocyte activation gene-3 (LAG-3), a CD4 homolog that binds MHC class II is yet another molecule that has been described to distinguish a unique sub-population of CD4+Foxp3+ Treg cells that expand at tumor sites (51). This study analyzed the frequency and phenotype of Foxp3+ cells in melanoma and colorectal cancer patients at different stages of disease and discovered that increased percentages of LAG-3-expressing Foxp3+ Treg cells preferentially expanded in the peripheral blood and tumor sites raising the notion that these cells represent a subset of tumor-iTreg cells (51). Other studies identified TNFR2, TIM-3, and ICOS as upregulated on Treg cells at tumor sites suggesting they may represent a distinct Treg cell subset that are generated specifically in response to TAA (52–54, 56). In a human melanoma study, for example, CD4+Foxp3+ Treg cells infiltrating tumor tissue not only displayed upregulated expression of ICOS but also exhibited a more potent suppressive activity compared to those derived from circulating blood cells (54). While these assessments were not particularly geared toward separating tumor-infiltrating Treg cells into natural or induced subset, it could be insightful if their expression patterns are considered in tandem with analysis focused at determining the composition of tumor-Treg cells with respect to their origin. (See Table 1 for a number of cancer studies in which some of these markers or TCR repertoire pattern were implicated in the suggested origin of tumor-infiltrating Treg cells.)

**Table 1:** **Natural and induced Treg cells in cancer**.

Species	Cancer type/tumor model	Treg phenotype	Suggested origin	Origin indicator	Reference
Human	Ovarian carcinoma	CD4+FOXP3+; CXCR3+, T-bet+	Natural	Helios expression	(41 )
Human	Colon adenocarcinoma	CD4+FOXP3+; CCR4+CTLA-4hi	Unknown		(89 )
Human	Ovarian cancer	CD4+FOXP3+; Helios+, CCR4^lo^	Unknown		(61 )
Mice/Rats	Colon carcinoma/melanoma	CD4+CD25+/Foxp3+	Natural	Expansion via mDC-TGF-β	(18 )
Mice	Fibrosarcoma	CD4+Foxp3+	Natural	Distinct TCR repertoire versus CD4+CD25−	(17 )
Mice	Colon carcinoma	CD4+Foxp3+	Induced	Foxp3 induction in CD4+CD25−	(20 )
Mice	Renal cell carcinoma	CD4+CD25+/Foxp3+	Induced	Foxp3 induction via TGF-β	(19 )
Human	Renal cell carcinoma	CD4+FOXP3+	Natural	Helios expression	(40 )
Mice, human	Glioblastoma	CD4+Foxp3+	Natural	Helios expression	(42 )
Mice	Colon adenocarcinoma	CD4+Foxp3+; Nrp-1^lo^, Helios^lo^	Induced	Helios and Nrp-1 expression	(43 )
Mice	Tumor cell line/melanoma	CD4+Foxp3+	Natural	Distinct TCR sequence versus CD4+CD25−	(70 )
Human	Hodgkin lymphoma	CD4+IL-10+ Tr1 and CD4+CD25+	Unknown		(26 )
Human	Ovarian cancer	CD4+CD25−FOXP3−IL-10+ Tr1 and CD4+CD25+Foxp3+	Induced and natural	IL-10 production or Foxp3 status	(30 )

## nTreg Versus iTreg in Tumors; A Function of Activation/Differentiation Status?

Perhaps, a healthy dose of objectivity is ideal in our trying to piece together the different phenotypes exhibited by Foxp3+ Treg cells in different tumors and finding a unifying phenotype that specifically identifies subsets. The increased expression of some of the afore-mentioned molecules upon T cell activation (57, 58) raises the possibility that the various unique phenotypes as observed in many tumor models and human cancers may simply represent an activation state and not an indication of a different cohort of iTreg cells generated from peripheral non-Treg cells. For instance, a recent study reported that the expression of GARP identifies activated human CD4+Foxp3+ Treg cells especially upon *in vitro* stimulation (58). Although very few studies have demonstrated the antigen specificity of tumor-infiltrating Treg cells (59, 60), one might speculate that the bulk of the Treg cells infiltrating the tumor have encountered and been activated by some TAA, hence are antigen-experienced. Therefore, it remains plausible that the different phenotypes as observed in different tumor models and human cancers is a reflection of their activation status and a factor of antigen repertoire to which the Treg cells are exposed in the tumor and/or draining lymph nodes. In sync with this notion, a recent study in late stage ovarian cancer patients noted a dominant population of Helios+ activated Treg cells in disseminated tumors (61). Another issue is whether the expression of these molecules signals a terminal differentiation stage. We previously reported that in humans, CD45RA-Foxp3hi cells are activated and terminally differentiated (62). In a murine study, KLRG1-expressing Treg cells were identified and also deemed to be terminally differentiated (63). Thus, tumor-infiltrating Treg cells may well be derived from pre-existing pool of peripheral nTreg cells but exhibit unique phenotypic properties reflective of their activation status and/or differentiation stage as opposed to being generated from non-Treg precursors, hence induced.

Expanding on this issue, it has been said that tumor-infiltrating Treg cells appear to display an effector phenotype that likely emanates from chronic exposure to TAA (10, 64, 65). Could expression of an effector phenotype distinguish between nTregs from iTregs? This is unlikely given that both potentially co-inhabit the tumor and are subjected to similar antigenic cues. Cretney et al., opined that activated/effector Treg cells display unique phenotypic features that distinguishes them from naïve cells (66, 67). In one of their studies, they described a distinct population of Blimp-1-expressing Treg cells with an effector phenotype (67). Given that IL-2 and inflammatory signals was shown to facilitate their production, one might speculate that the prevalence of such inflammatory cytokines/signals in the tumor surroundings may favor the recruitment or generation of these functionally mature effector Treg cells. In this context, Blimp-1 could be useful to identify effector Tregs which are derived from the natural pool versus those induced from CD25− cells *in situ*. Perhaps, an evaluation of a plethora of activation-associated markers such as described by Cretney and colleagues may yield some clues as to which subset of tumor-infiltrating Treg cells are natural or induced regardless of their antigen experience.

At the genetic level, molecular analysis has revealed that while nTreg cells show a stable hypomethylation pattern at the Foxp3 locus, iTregs generated *in vitro* and *in vivo* are fickle, presenting with unstable Foxp3 expression with partial hypomethylation pattern (68, 69). Although both iTreg and nTreg in the tumor may be indistinguishable in terms of having an effector phenotype, assessing Foxp3 epigenetic modification patterns could be useful to differentiate nTregs from iTregs.

## TCR Repertoire Diversity and Antigen Specificity of Tumor-Infiltrating Treg Cells

Currently, there is paucity of data addressing the issue of antigen specificity and TCR repertoire within tumor-associated Treg cells and how this information may define induced versus nTreg cells. The notion that Treg cells accumulating within tumors might be nTreg cells was presented by Gallimore’s lab. In one of their studies as mentioned previously, they analyzed the TCR repertoires of Treg cells and T conventional cells within the tumor tissue and found that they were largely distinct concluding that based on this finding, tumor-Tregs are likely derivatives of nTregs (17). In another study using non-TCR-transgenic mice, immunoscope-based analysis of the TCR repertoire of tumor-infiltrating Treg cells and T effector cells revealed that each population exhibited a skewed and distinct repertoire indicative of clonal expansion, hinting that the tumor-infiltrating Tregs are likely a few clones that proliferate extensively in the tumor (70). Further analysis of CDR3 sequences revealed some public sequences that were unique to Treg cells obtained from multiple tumor tissues but had little overlap with T effector cells arguing against the possibility that the Treg cells were converted from T effector cells, although based on the limited scope of the work, such possibility still cannot be excluded.

Treg cells are selected with TCRs specific for self peptide: MHC constituents (71, 72) and many TAA are self antigens (73). Furthermore, Treg cells can recognize an array of tumor-associated immunogenic self antigens (74, 75). So, it is possible that tumor-infiltrating Treg cells exhibit unique TCR repertoire highly reactive against some of the TAA. Supporting this notion, a human-melanoma-infiltrating Treg clone specific for LAGE-1, a cancer/testis antigen that is expressed in many types of tumors was identified in a study (76). It should be reiterated here that the expression of cancer/testis antigens is normally restricted to male germ cells but not in adult somatic tissues. On that account, they are cancer tissue-specific self antigens. In another study, the same group reported the establishment of CD4+ Treg clones generated from tumor-infiltrating lymphocytes of cancer patients which were reactive against another tumor-derived ARTC1 peptide (77). In another unrelated study, NY-ESO-1 (New York esophageal squamous-cell carcinoma-1)-specific CD4+ T cells were generated from naïve T cell preparation upon Treg cell depletion suggesting that Treg cells, presumably an antigen-specific subset suppressed NY-ESO-1-specific T cell induction in cancer patients (78). Thus, circulating tumor-antigen-specific Treg cells exist at least in patients with certain cancers (79). While these studies suggest to a certain extent, the self specificity of tumor-infiltrating Treg cells, the issue of their origin was not addressed. How might iTreg cells and nTreg cells in the tumor differ with respect to their antigen specificity and repertoire? Answering this question requires a clear understanding of which of these two subsets predominates in specific cancers. Then, our efforts could expand to deciphering their peptide specificity, immunodominant epitopes of such peptides, and TCR diversity of Treg cells that may recognize them through combination of techniques including but not limited to cloning, proteomics, and spectratyping analysis.

## Tumor-Treg Cell Recruitment and Trafficking

The recruitment of Treg cells (natural or induced) into tumors likely involves complex, multi-step processes that ultimately culminate in the high frequencies observed in many cancers. Perhaps, the expression of certain receptors may be key to unraveling some of these processes and sorting the suppressor cells. One potential candidate protein is Neuropilin-1 (Nrp-1), the expression of which was found to be low on *in vivo*-generated iTreg cells under non-inflammatory conditions unlike nTreg cells which preferentially expressed this protein at high levels (43). In tumor-bearing mice, Nrp-1 expression on Treg cells was demonstrated to promote their recruitment to tumor site via tumor-derived VEGF gradient (80). Anecdotally, Nrp-1, the expression of which is very low in naïve T conventional cells is under Foxp3 control as ectopic expression of Foxp3 in these cells led to induction of Nrp-1 (37, 81). Given that TGF-β can bind Nrp-1 in addition to inducing Foxp3 expression (35, 82), it remains plausible that TGF-β-induced Foxp3+ iTreg cells, armed with Foxp3-induced Nrp-1 expression, respond to further TGF-β binding in a positive feedback loop, and ultimately become recruited across similar gradient as the nTreg cells.

Chemokine receptor pattern while largely unexplored, could be another critical aspect of tumor-affiliated Treg cells that could be useful in determining Tumor-Treg sub-groups. For example, in human ovarian carcinomas, selective accumulation of Treg cells expressing high levels of chemokine receptor CXCR3 was noted (41). Similarly, Treg cells that infiltrated colorectal tumor mass preferentially expressed CCR6 which appeared to promote their recruitment via tumor-associated macrophage production of CCL20 (83). In skin tumor-bearing mice, CCR5 was preferentially expressed on tumor-infiltrating Treg cells, which seemed to be recruited to the tumor via its ligands, CCL3, 4, and 5 that was produced by myeloid-derived suppressor cells (MDSCs) (84). Similarly, CCR5 signaling appeared to facilitate the recruitment of Treg cells to pancreatic adenocarcinoma (85). Other chemokine receptors implicated in Treg trafficking to tumor sites include CXCR4, which drives Treg cells toward tumor site via interactions with CXCL12 that is produced within the tumor microenvironment, as well as CCR8 and CCR10 (86–88). In the case of CCR10, hypoxia within ovarian tumor environment promotes the secretion of CCL28 by cancer cells which in turn enhances the recruitment of Foxp3+ Treg cells via CCR10 (87). Furthermore, in studies of oral squamous-cell carcinoma and colon adenocarcinoma, increased frequencies of tumor-associated CCR4hi cells were reported (89, 90). Consistent with this and other reports (16, 91, 92), we have recently identified CCR4 to be highly expressed on the majority of tumor-infiltrating Treg cells in a human melanoma study (manuscript in preparation). Notably, their phenotype was unique and distinct from their counterparts in non-tumor-associated peripheral blood. Whether these Treg cells are peripherally recruited by tumor-derived factors such as CCL22, which is a chemokine that is widely produced by a number of tumors, and a ligand for CCR4 (12, 65) remains to be determined and is a subject of our ongoing investigations.

In contrast to our observations and that of others mentioned above, one report found that tumor-infiltrating Treg cells exhibited markedly reduced levels of CCR4 in HNSCC relative to circulating Tregs (61). One obvious explanation for variabilities between these studies is that differences in tumor type, infiltrating immune cells, and stage of disease likely impacts the phenotype of Treg cells prevalent within tumors at time of investigation. Despite the lack of any extrapolation from all these studies as to the natural or induced status of tumor-Treg cells, they bring to light, the notion that the tumor milieu likely shapes the composition of Treg cells present within it as different Treg cell subsets express different homing receptors based on the environmental cues to which they are subjected (93). Thus, different tumors may exhibit distinct Treg cell composition that reflects such properties. In this regard, evaluation of homing receptor expression pattern in various human cancers may thus shed more light to whether they are locally induced, or are expanded from a recruited natural population.

## Induced/Adaptive Treg Generation in Tumors

The mechanisms involved in *de novo* generation of adaptive Treg cells are still unclear. Several lines of evidence point to the suppressive cytokine milieu prevalent within the tumor environment as a major contributory factor (94). For instance, TGF-β can induce iTreg cells and it is well established that several tumor lines utilized in murine tumor studies secrete TGF-β (19, 95–97). Other tumor-derived soluble factors such as GM-CSF and VEGF may recruit or expand MDSCs which then secrete cytokines that could potentially induce Treg cells (98, 99). Additionally, tumor-associated macrophages or DCs may be instrumental in inducing Treg cells or recruiting discrete subsets of Treg cells with distinct phenotypes (83, 100).

Similar to the phenomenon of infectious tolerance (101), Treg cells may also directly enlist naïve T cells into the regulatory pool. In this regard, Treg cell production of IL-10 and TGF-β (102, 103) may also modulate some naïve CD4+ T cells, converting them to cells with inhibitory function. Another possibility is an indirect effect via modulation of DCs. Treg cells via CTLA-4 may keep DCs in an immature state by engaging CD80 and CD86 molecules on these antigen presenting cells (102). Such immature DCs may induce Foxp3 or Foxp+-like phenotype, in line with their demonstrated ability to efficiently induce iTreg cells *in vivo* (104). The modification of tumor-associated APCs is however not restricted to Treg effect alone. Other inhibitory agents produced by tumors such as IDO (105) may re-shape DCs to become tolerogenic and in turn promote induction of Foxp3+ Treg cells (106). Taken together, adaptive Treg cell generation may be promoted by tumor-related expression of key cytokines and soluble factors that have the potential to induce Foxp3+ cells from existing pool of tumor-infiltrating conventional CD4+ T cells or recruit discrete regulatory CD4+ T cells from distal sites.

**Figure 1:**
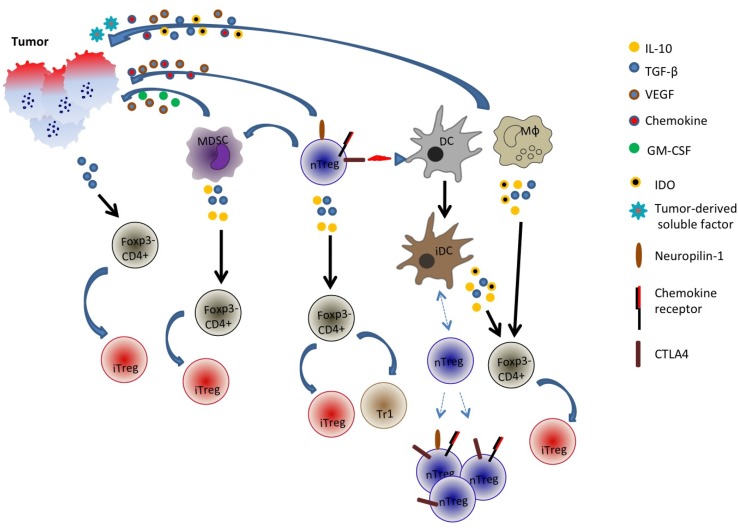
**Generation and recruitment of adaptive/induced Tregs in the tumor microenvironment**. Tumor cells may secrete an array of cytokines and soluble factors that facilitate the induction of Foxp3 in Foxp3− cells or the recruitment of multiple cell types including natural Treg cells, myeloid-derived suppressor cells (MDSC), dendritic cells (DC), and macrophages. These cells in turn may secrete inhibitory and immune-suppressive factors such as TGF-β, IL-10, and indoleamine 2,3-dioxygenase (IDO) that could potentially convert some Foxp3− CD4+ cells into Foxp3+ cells. Additionally, tumor-derived factors or Treg interaction with DCs may promote generation of tolerogenic or immature DC (iDC) that recruit distinct populations of natural Tregs. nTreg is CD4+Foxp3+ cells while iTreg is CD4+Foxp3 variable.

In a nutshell, it is evident that the generation of adaptive Treg cells is likely a complex phenomenon and multiple pathways may be involved (Figure 1). Adding to this complexity is the tumor itself: its properties such as cytokine and chemokine milieu, angiogenic capabilities, etc. may determine or shape the generation of these peripherally induced adaptive Treg cells.

## Foxp3 Stability as an Indicator of Natural Versus Induced Treg Cells in Tumors?

Addressing the issue of Foxp3 stability within tumor-associated Treg cells, a recent report evaluated tumor-resident Treg cells. Using reporter mice that bear melanoma, authors were able to differentiate between “ex” and “current” Foxp3+ Treg cells (64). In this study, it was found that majority of the tumor-Treg cells retain Foxp3 expression and only a minor population lost its expression providing evidence that Foxp3 expression even in an inflammatory environment as the tumor remained stable. Since iTregs only show a partial DNA hypomethylation pattern unlike nTregs (68, 69), indicating a transient opening up of the Foxp3 locus, they do not to stably express Foxp3 and may even likely lose its expression in the absence of signals that elicited Foxp3 induction. Extrapolating from this, it is tempting to conclude that majority of tumor-Treg cells are likely nTregs based on their Foxp3 stability and not iTregs as Foxp3 unstable Treg cells would otherwise constitute a sizable fraction of tumor-Tregs if they were induced from conventional CD4+ T cells. Evaluations such as genetic profiling of Foxp3 locus thus may be useful in delineating what constituency Treg cells in different tumors belong to, i.e., the “i” or the “n” family.

## Function of Natural Versus Induced Treg Cells

Several questions linger as we attempt to understand the role of iTreg cells versus nTreg cells in tumor immunobiology: is the role of iTregs largely redundant when nTreg cells are present? If not, do they possess similar specificity and or play similar roles as their natural counterparts? Two studies, one in a colitis model, the other in Foxp3-deficient mice, which succumb to lymphoproliferative disease, demonstrated that full protection from disease was only achieved when both nTreg cells and iTreg cells were present, suggesting that the function of each Treg cell group is complementary (49, 107). As Lafaille and colleagues surmised, a division of labor between nTreg cells and iTreg cells seems a plausible arrangement as far as their functional roles in regulating immune responses (13). One might speculate that given their sheer dominance and omnipresence, nTreg cells share the greater bulk of curtailing T cell responses while adaptive Treg cell contribution is solicited as needed and differs on a case-by-case à la cancer-by-cancer model. Relating to this principle, a study described the accumulation of nTreg cells and iTreg cells in the tumor microenvironment, with the latter possessing TCR specificity for a defined antigen expressed by the tumor. Suppression by cognate-antigen-specific iTreg cells was restricted to CD4+ T cells and occurred only within the local tumor environment while suppression of CD8+ T-cell response was independent of these tumor-antigen-specific iTreg cells (108). From this, one might deduce that iTreg cells evolve peripherally as in the tumor only to control some arms of the immune response while the nTreg cells control others.

In many colorectal cancer studies, the observation that increased Foxp3+ Treg cells correlate with good prognosis is particularly intriguing (109). An argument has been made that the Treg cells in this context may largely be involved in controlling potential inflammation that could ensue in response against the commensal bacteria present in the lower intestine if Tregs are absent (13). Given that GALT environment is permissive for induction of iTreg cells, it is tempting to speculate that the FOXP3+ Treg cells in colorectal cancer are mostly iTreg cells. To test this possibility, phenotypic characterization, TCR repertoire analysis, and FOXP3 methylation status of Treg cells in colorectal tumor tissues in parallel with solid tumors from sites not heavily associated with intestinal commensal bacteria could be a starting point.

Summarily, elucidating what environmental and molecular cues facilitate the generation of iTreg cells and the type of role they play particularly in various cancers would be eye-opening and may pave way for manipulating the immune system to prevent their generation in such context. At any rate, more studies are warranted to tease out who does what and to what degree is this division of labor shared.

## Treg Therapy: Targeting Natural and Adaptive/Induced Tregs

To prime and/or boost anti-tumor immune response, selective removal or reduction of Treg cells have been carried out in a number of murine tumor studies (12). This depletion is generally achieved via the use of anti-CD25 mAb (PC61), anti-FR4 mAb, and diphtheria toxin, the latter to DEREG mice (which express diphtheria toxin receptor under the control of Foxp3 promoter (110–114). In humans, daclizumab (anti-CD25) and denileukin diftitox (ONTAK, a fusion protein of diphtheria toxin and recombinant human IL-2) treatment has also shown some efficacy in some cancers, consequent to their Treg cell depletion effect although with varying degrees of success (10, 115). Cyclophosphamide, a chemotherapy agent that is a part of treatment regimen in some cancers is also known to target Treg cells by reducing their frequencies or function (116–119). In combination with tumor vaccination, all three agents were tested in melanoma patients in one study. Interestingly, only modest reduction in Treg cells (as determined by methylation status of FOXP3 intron 1 within Treg cells) was noted in the peripheral blood of patients in the treatment groups (120). In a recent clinical trial utilizing multiple tumor-associated peptides as a therapeutic vaccine for renal cell cancer, T-cell responses of treated patients were associated with better disease control and correlated with lower numbers of FOXP3+ Treg cells prior to vaccination. This revelation prompted the incorporation of cyclophosphamide to the vaccine regimen in subsequent study which demonstrated that reduced Treg cell numbers achieved by this approach further improved patients’ immune responses to the tumor antigens and importantly, their overall survival (121). The caveat to all these studies is that the effect of these Treg cell depletion/reduction protocols have not been evaluated on Treg cell subsets and essentially no information is available on whether iTreg cells are more susceptible to these regimen than nTreg cells or vice versa. Thus, critical evaluation of the residual Treg cell fractions not targeted by these agents is warranted as they may represent an induced population with phenotypic changes that make them evade depletion regimen.

On the other hand, there is some evidence that nTreg cells are more resistant to oxidative stress or apoptosis than conventional T cells (122). Based on this, nTreg cells, assuming they account for the majority of tumor-infiltrating Treg cells, may be the subset that is more resilient to therapeutic modalities aimed at eliminating tumor-Tregs. In this regard, multi-pronged approach combining multiple agents targeting “i” and “n” Tregs may be necessary to achieve efficient elimination. While their differential expression is yet to be assigned to either iTreg or nTregs cells, CCR4, PD-1, and CTLA-4, which have been shown to be highly expressed on tumor-Treg cells (123) offer potential targets for treatment of cancers enriched in Treg cells with such phenotype. In alignment with this line of thinking, the combination of anti-CTLA-4 and anti-PD-1 antibody treatment in a mouse B16 melanoma study led to substantial reduction in Treg cells as well as myeloid cells with a concomitant increase in tumor-infiltrating effector T cells (124). Agonist antibody against Glucocorticoid-induced tumor necrosis factor receptor family-related protein (GITR), also expressed on Treg cells (125), is another treatment route that holds promise. In a murine model of melanoma, its administration promoted potent anti-tumor immune response (126). Similarly, in combination with anti-CTLA-4 antibody, anti-GITR administration evoked regression of established fibrosarcoma and colon carcinoma in other studies (127, 128). In either case, the positive outcomes were ascribed to anti-GITR antibody-mediated attenuation of Treg function or decreased intra-tumoral Treg cell accumulation, in addition to augmented CD+ T-cell effector response (126–128). For advanced melanoma, it is worth mentioning that administration of humanized anti-CTLA-4, ipilimumab improved survival of patients with metastatic melanoma in a clinical trial (129). In our recent investigations, we found that tumor-infiltrating T cells contained a higher frequency of effector Tregs with activated phenotypes compared with peripheral blood. Correspondingly, Tregs with a naive phenotype were barely detected in tumors while peripheral blood contained both naïve and effector Tregs. These tumor-infiltrating effector Tregs dominantly expressed CCR4, proposing CCR4 as a possible target for Treg control (manuscript in preparation).

The finding that human adaptive CD4+FOXP3+ Treg cells which express CD39, and CD73, and produce adenosine was described by Whiteside and co-workers (130). They demonstrated *in vitro*, the generation of iTreg cells with similar phenotype (except for FOXP3) in co-cultures simulating some of the features unique to the human cancer in which equivalent Treg cells were observed (131). They found that both adenosine and PGE2 produced by these iTreg cells co-operate in mounting strong suppressive function against autologous T effector cells. Thus, Whiteside proposed that targeting adaptive Treg cells by interfering with adenosinergic pathways and PGE2 production could be a viable therapeutic platform to disarm iTreg cells in human cancers (132).

Lastly, methods aimed at disrupting iTreg cell induction such as interfering with TFG-β signaling in relevant tumors could be complementary approaches to vaccination. Using siRNA-mediated downregulation of TGF-β production by B16 melanoma cells, this idea was explored by Mills and colleagues and they reported that tumor growth was hampered (133). This coincided with reduced tumor-Treg cell numbers although it was not clear as to whether this reduction affected iTreg cells as we might postulate based on experimental design.

Worth mentioning is the issue of Treg function at the interface of autoimmunity and cancer. The pivotal and positive role of Treg cells is exemplified in mice as well as IPEX patients in which impaired Foxp3+ Treg cell development culminates in wholesale breakdown of immune tolerance (1, 134, 135). When placed in the context of tumors however, Treg suppressive function appears for the most part, to result in unfavorable prognosis. In fact, studies that portray Treg presence within the tumor in a bad light, i.e., inhibiting anti-tumor response outweigh those demonstrating they may have favorable contributions in cancer (10–12). In a recent report, melanoma patients who had better response following treatment with high dose IL-2 plus vaccine had higher Treg frequencies portraying a correlation between Tregs and better response against tumor (136). Thus, therapeutic strategies that are focused on Treg reduction in order to promote tumor clearance need to take this apparent duality in Treg function into account. More importantly is the effect such depletion may have on elevating a patient’s risk for developing autoimmune conditions especially if systemic Treg depleting routes are utilized. In this regard, localized Treg reduction by intratumoral administration of Treg depleting agents which has shown efficacy at reducing tumor burden in mice (127) may offer a more favorable treatment platform without the inherent risk of the global Treg elimination assuming the tumor is accessible. Furthermore, since Treg cells in tumor environment appear to be of the effector Treg phenotype and may exhibit augmented suppressive activity when compared to those in circulation (64, 137–139), localized Treg modulation approach could be a viable option to target only a subset of highly suppressive, effector Treg cells based on specific molecules which they uniquely upregulate in response to tumor antigens. By so doing, the bulk of nTreg cells are left intact while only those “in action” are removed. This should be a feasible approach as we have recently tested the effect of anti-CCR4 antibody on subsets of human Treg cells in melanoma patients and found it to efficiently eliminate a population of CCR4-expressing effector Tregs while sparing naïve Treg populations (manuscript in preparation). Until we have some evidence of the nature and extent of the contributions of nTregs and iTregs in various tumors, treading carefully on indiscriminate Treg depletion for cancer therapy however seems a reasonable proposition.

## Perspectives

Different subsets of Treg cells may be committed to regulate specific arms of immune responses (140). Understanding the functional capabilities of both iTreg cells and nTreg cells will no doubt help in guiding future treatment platforms. A number of possibilities exist: their elimination from the tumor microenvironment, blocking their ability to produce a number of immune-suppressive/immune-altering molecules such as adenosine, PGE2, perforin, and granzyme B, targeting anti-apoptotic pathways, disrupting their ability to proliferate and or persist in tumors, etc. The list is not conclusive as our understanding continues to expand about the nature of Treg cells that prevail in different cancer types. Thus, additional investigations are necessary to first determine whether the variabilities seen among different cancer studies with respect to phenotype associated with the tumor-Treg cells relate to their origin, i.e., are they natural or peripherally iTreg cells. From such information, we may be able to optimize Treg cell-targeted approaches to reduce or eliminate not just a major subset that is prevalent within the tumor, but a minor subset that could contribute to hindering optimal therapeutic success in the settings where their presence is related to poor survival. To this end, designing antibodies against some of the molecules that appear to preferentially mark Treg cells infiltrating tumors may be a good investigational direction worth pursuing in our quest to treat cancers. It will be interesting to see whether such studies reveal information about the effect of treatment on subsets of Treg cells that are affected, and those that are resistant to modulation. At any rate, treatment modalities focused on elimination of Tregs or disruption of their function to bolster anti-tumor immunity should take into account the differences between cancer types, the subset of the Tregs that predominate within the tumor, and their recruitment dynamics.

## Conflict of Interest Statement

The authors declare that the research was conducted in the absence of any commercial or financial relationships that could be construed as a potential conflict of interest.
